# A comprehensive dataset of animal-associated sarbecoviruses

**DOI:** 10.1038/s41597-023-02558-5

**Published:** 2023-10-07

**Authors:** Bo Liu, Peng Zhao, Panpan Xu, Yelin Han, Yuyang Wang, Lihong Chen, Zhiqiang Wu, Jian Yang

**Affiliations:** NHC Key Laboratory of Systems Biology of Pathogens, Institute of Pathogen Biology, Chinese Academy of Medical Sciences and Peking Union Medical College, Beijing, 110730 China

**Keywords:** Databases, Virology

## Abstract

Zoonotic spillover of sarbecoviruses (SarbeCoVs) from non-human animals to humans under natural conditions has led to two large-scale pandemics, the severe acute respiratory syndrome (SARS) pandemic in 2003 and the ongoing COVID-19 pandemic. Knowledge of the genetic diversity, geographical distribution, and host specificity of SarbeCoVs is therefore of interest for pandemic surveillance and origin tracing of SARS-CoV and SARS-CoV-2. This study presents a comprehensive repository of publicly available animal-associated SarbeCoVs, covering 1,535 viruses identified from 63 animal species distributed in 43 countries worldwide (as of February 14,2023). Relevant meta-information, such as host species, sampling time and location, was manually curated and included in the dataset to facilitate further research on the potential patterns of viral diversity and ecological characteristics. In addition, the dataset also provides well-annotated sequence sets of receptor-binding domains (RBDs) and receptor-binding motifs (RBMs) for the scientific community to highlight the potential determinants of successful cross-species transmission that could be aid in risk estimation and strategic design for future emerging infectious disease control and prevention.

## Background & Summary

Coronaviruses (CoVs) are a group of enveloped viruses belonging to Coronaviridae and currently contain four known genera, Alpha-, Beta-, Gamma-, and Delta-CoVs, that vary in their distribution, host species, and pathogenicity to humans. Sarbecovirus (SarbeCoV), a subgenus within Beta-CoV, has resulted in the emergence of the highly pathogenic human viruses SARS-CoV and SARS-CoV-2. SARS-CoV caused more than 8000 confirmed cases in 2002–2003^1^, whereas SARS-CoV-2, a causative agent of COVID-19, has rapidly infected the global population with over 762 million confirmed cases (https://covid19.who.int/), and remains a significant threat to global health and the economy. Furthermore, evidence suggests that SarbeCoVs have high recombination and mutation rates, allowing them to infect and survive in different hosts worldwide^2^. Thus, recent research has intensely focused on surveys of SarbeCoVs carried by susceptible animals to enhance our knowledge of viral diversity, host specificity, and geographical distribution.

The origins of SARS-CoV and SARS-CoV-2 remain controversial due to the remaining genomic differences^3^. It is generally been thought that both SARS-CoV and SARS-CoV-2 originated in bats, and zoonotic spillover to humans has likely occurred through one or more intermediate hosts. Recently, several studies have shown that potential intermediate hosts may include Malayan pangolins, rabbits, ferrets, foxes, raccoons and dogs because the spike (S) protein of SARS-CoV and SARS-CoV-2 is capable of binding to their angiotensin-converting enzyme (ACE2), which facilitates virus entry^1,4^. In addition, molecular and serological evidence has revealed the reverse zoonotic potential of SARS-CoV-2 infection in several pets and domestic animals from different countries. Zoo tigers, lions, snow leopards, and pumas and domestic cats, dogs and minks have been confirmed to naturally acquire SARS-CoV-2 infection^5^. Despite no conclusive evidence that domestic animals can actively spill back SARS-CoV-2 to humans, the potential human-animal-human transmission cycle needs to be recognized and further investigated^6^. Thus, a better understanding of existing viral populations along with their ecological characteristics would be of importance for detecting potential interspecies spillover^7^.

Given that molecular techniques are widely applied in the identification and functional analyses of viruses, comprehensive retrieval of the virus sequences along with related meta-information facilitates in-depth research on the origin and evolution of SARS-CoV and SARS-CoV-2 among different animal hosts. However, relevant information such as virus sequence, host species, sampling time, and location has not been uniformly recorded and is only sporadically available in GenBank records or related literature. Furthermore, the considerable number of human-derived SarbeCoVs undoubtedly complicates the screening process of animal-associated SarbeCoVs from the public domain. Therefore, we established a sequence-centric dataset for the curation of related meta-information of animal-associated SarbeCoVs. Thus far, this dataset contains 1,535 SarbeCoVs identified from 63 different animal species globally.

## Methods

### Data collection

**Fig. 1:**
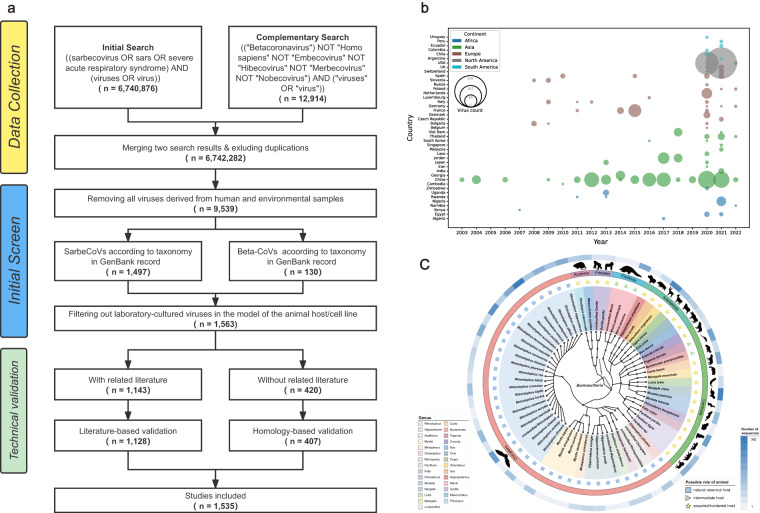
Schematic diagram of data curation. (**a**) Flow diagram of the data search, screening and validation process. (**b**) Bubble chart of the annual and geographical distributions of presently identified animal-associated SarbeCoVs. The area of each bubble correlates with the number of SarbeCoVs. (**c**) Overview of the classification, from host genus to order, of animal-associated SarbeCoVs included in this study. The label “possible role of animals” refers to the potential role that animals are currently known to play as natural reservoirs, intermediate, incidental hosts in the circulation of SarbeCoV. The presence of potential natural reservoirs, intermediate hosts and incidental hosts is marked by a colored blocks, triangles, and pentagrams respectively. The outer blue ring represents viral counts that are normalized by sequence number on a log scale.

The data collection and inclusion procedures are outlined in Fig. 1a. To retrieve all known sequences from the public domain, an initial search within GenBank^8^ was performed using the keywords (“Sarbecovirus” OR “SARS” OR “Severe acute respiratory syndrome”) AND (“viruses” OR “virus”). A total of 6,740,876 GenBank records that matched the above keywords were retrieved and stored in a local system (as of February 14,2023). Despite conducting an exhaustive search, there is no guarantee that all records were collected. The possibility of missed SarbeCoVs may be inevitable in certain cases due to misclassification of sarbecoviruses by the submitters or historical changes in taxonomy. For instance, the term sarbecovirus was proposed as a novel subgenus within the genus Beta-CoV according to the ICTV demarcation criteria in 2017. Before that, some of the identified SARS-related coronaviruses (SARSr-CoVs) may have been categorized into the unclassified Beta-CoV in the NCBI taxonomy database. To ensure the integrity of data collection, a complementary search using the additional keywords (“Betacoronavirus” NOT “Homo sapiens” NOT “Embecovirus” NOT “Hibecovirus” NOT “Merbecovirus” NOT “Nobecovirus”) AND (“viruses” OR “virus”) was conducted with specific attention given to collect unclassified Beta-CoVs that might belong to SarbeCoVs. After removing the duplicates from the two search results, there were 6,742,282 records possibly associated with SarbeCoVs that needed further review.

Then, relevant information on each virus, such as its sequence, classification in the virus taxonomy, host species, sampling time, sampling location, detection method (e.g., PCR, metagenomics), specimen type (e.g., tissue, cell line), etc., was extracted from GenBank records using in-house BioPerl scripts. Since this study focuses on naturally transmitted animal-associated SarbeCoVs that are particularly relevant to the emergence of zoonotic infectious diseases^9^, unrelated records were carefully excluded by considering the following three criteria: (i.) all viruses derived from humans and environmental samples were removed; (ii.) all viruses that were not classified as a sarbecovirus or betacoronavirus were excluded; and (iii.) all viruses isolated from non-animal samples but laboratory-cultivated in the animal host model or cell line were filtered out. Herein, 1,563 virus records were pre-collected in the dataset after initial curation. To ensure the accuracy of meta-information, we further conducted an intensive double-check for published viruses based on data reported in the related literature and the taxonomy database of the NCBI, with an emphasis on supplementing missing data and clarifying ambiguous data.

### Data curation

As the dataset integrates information spanning 20 years (Fig. 1b), refining data entries with a consistent and controlled vocabulary was essential to ensure that the same scientific notation, which may have been noted differently by the submitter, was assigned the same unique terms. In this study, three general types of meta-information collected from different sources needed to be uniform before being entered into the dataset. First, all host information reported in this study underwent intensive review to avoid possible errors in taxonomic classifications (Fig. 1c). The names of animal hosts were standardized using the taxonomy database of the NCBI^10^, and species names that could not be confirmed were excluded. In some instances, the common names of host species were uniformly converted into scientific names using binomial nomenclature. Second, all available location information for the records was categorized into four geographical and administrative levels (i.e., continent, country, subregion and Global Positioning System coordinates). Related latitude and longitude were transformed into decimal format using the website (https://www.gps-coordinates.net). Third, certain studies have conducted long-term surveillance on susceptible animals and reported a batch of viruses. However, these studies provided only a period of time without specifying the sampling time for each individual sample. To address this issue, we defined two fields, “date from” and “date to”, which served as the starting and ending dates, respectively, for all viruses identified in the same surveillance program.

### RBD & RBM extraction

The receptor-binding domain (RBD) is located in the S protein and plays a crucial role in facilitating virus entry into host cells, as well as in regulating viral infectivity, pathogenesis, and host range. Evidence has shown that the RBD contains a critical receptor-binding motif (RBM), which binds to the outer surface of the claw-like structure of host ACE2^11^. Certain amino acids at specific positions can increase the affinity with host ACE2^12^. To extract sequences of the RBD and RBM, we first performed multiple sequence alignment to align all sequences of the SarbeCoV S gene (if present). Subsequently, we used SARS-CoV-2 (GenBank accession: NC_045512) as the reference genome to annotate the existing RBD and RBM regions. Following the exclusion of all RBD and RBM sequences with ambiguous bases (Ns), we collated a total of 726 RBD and 750 RBM amino acid sequences.

## Data Records

The dataset is publicly accessible online via Figshare^13^ and consists of three sequence sets, the available sequence of each virus and sequences of RBD and RBM (if present). In addition, the meta-information available on animal-associated SarbeCoVs was curated into 25 fields that were categorized into six groups as follows:

- Virus: Description of basic sequence information of the respective SarbeCoVs that includes six fields, namely, virus name, strain, accession, sequence description, sequence length, and completeness. The field “completeness” was assigned a label of “true” if a complete genome was available.
- Host: Description of the animal host from which the virus was derived, including three fields: host, taxonomy ID, and possible role of animals. The field “possible role of animals” refers to the potential role that animals are currently known to play as natural reservoirs, intermediate hosts or incidental hosts in the circulation of the SarbeCoV^5,6^.
- Sampling location: Description of the detailed location of the sample that includes four fields, namely, continent, country, subregion, and GPS coordinates.
- Sampling time: Description of the specific time at which the sample was collected, including three fields: namely, sampling date, date from, and date to. If the submitter did not provide any temporal information, then we assigned a label of “NA”.
- Preparation method: Description of the methods of sample preparation used to identify the SarbeCoV that includes three fields, namely, specimen type (e.g., oral swab, faeces, or tissues), cell line, and detection method (e.g., PCR or high-throughput sequencing).
- Reference: Description of the available literature that includes five fields: title, author, affiliation, publication (if available), and PubMed ID.

## Technical Validation

After initial curation, the dataset consisted of 1,563 SarbeCoV sequences, including 1,143 published sequences and 420 unpublished sequences (without related literature). In an attempt to ensure the accuracy and validity of sequences, two additional examination steps were implemented. The first step involved literature-based examination to identify any inconsistencies in virus taxonomy between GenBank records and related literature. Herein, a total of 1,128/1,143 virus sequences were verified to be associated with SarbeCoVs based on taxonomic information described in related literature, whereas 15 virus sequences (15/1,143) were associated with Alpha-CoVs rather than SarbeCoVs according to phylogenetic analysis in the literature. We excluded these 15 confirmed Alpha-CoVs and independently cross-checked them by two different team curators to ensure accuracy. The data source used to compile these published sequences is also cited in the manuscript^14–122^.

Nevertheless, the absence of peer-reviewed literature may pose an obstacle to further data validation. Therefore, we implemented a homology-based examination for unpublished sequences. All 420 unpublished sequences were aligned to the nonredundant nucleotide database (NT) using the BLAST suite of the NCBI. The taxonomic report generated from the BLAST result revealed that the majority of the unpublished sequences (404/420) shared an overall nucleotide similarity of >91% with currently confirmed SarbeCoVs. Additionally, a set of unpublished sequences (7/420) deposited by the same submitter shared only 83–89% similarity with SarbeCoVs, but further phylogenetic analysis demonstrated that they were closely related to bat coronavirus BM48-31/BGR/2008 (GenBank accession: NC014470). In contrast to the aforementioned sequences that showed the best matches with known SarbeCoVs, the last remaining unpublished sequences (9/420) were found to be highly homologous to Alpha-CoVs (>95% nucleotide similarity) rather than SarbeCoVs. As a result, we removed these 9 probably misclassified Alpha-CoVs from our dataset.

Despite the potential of the homology-based examination to verify the correlation of these unpublished sequences with SarbeCoVs, it remains challenging to discern whether they originated from infected or contaminated samples. For instance, it is known that amphibians are not naturally susceptible to SARS-CoV-2 infection based on our current knowledge. Without contextual clues, our curator lacked sufficient evidence to determine the transmission route of four SARS-CoV-2-related sequences (SC2r-CoVs) identified from Scincomorpha lizards in Nigeria (GenBank accession: ON564647-ON564650). However, these four sequences shared a nucleotide similarity of >99% with SARS-CoV-2, suggesting that they were probably derived from contaminated samples. Consequently, we empirically excluded four SC2r-CoVs obtained from lizards. Finally, we will continue trying to verify whether any relevant literature is available to solve possible inconsistencies in the follow-up study.

## Usage Notes

Users can summarize the current research efforts on animal-associated SarbeCoVs for individual investigation purposes and methodologies. However, it is worth noting that several large-scale surveillance programs on the screening of SARS-CoV-2 have used only serological detection methods. Related findings without available sequences will be excluded from this study. Consequently, the current data cannot exactly represent the total count of positive cases of SarbeCoVs carried by all animal hosts. In addition, despite our efforts to eliminate misclassified SarbeCoVs, it is still possible that some may remain in the dataset. Users should use caution in the biological interpretation of the statistical results generated in this study.

**Fig. 2:**
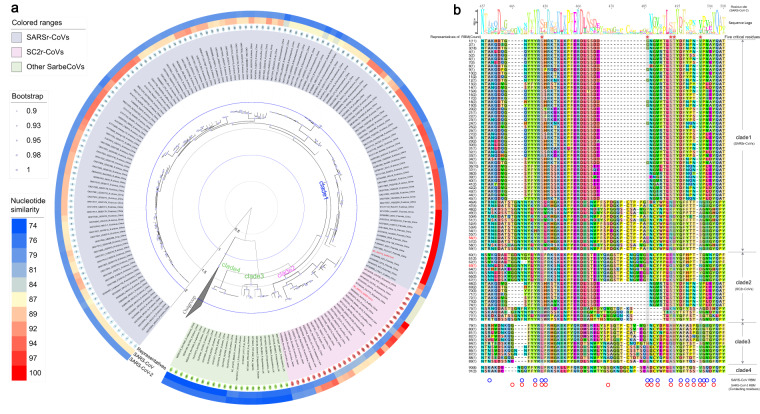
Characterization of complete S protein and RBM sequences in natural reservoirs/intermediate hosts of SarbeCoVs. (**a**) Phylogenetic tree based on the complete S sequences, accompanied by two homology-based heatmaps that represent the sequence similarity with SARS-CoV (middle ring) and SARS-CoV-2 (outer ring) at the whole-genome level. The SarbeCoVs carrying identical amino acid sequences of the RBM are labelled with the same serial number (inner ring). (**b**) Comparative sequence alignment of the representative RBMs with identical amino acids. The serial number of representatives is correlated with the sequence number shown on the phylogenetic tree (**a**). The five critical residues are highlighted with red pentagrams. Other contacting residues in the SARS-CoV and SARS-CoV-2 RBM that interact with hACE2^81,124^ are marked by blue and red circles, respectively.

Additionally, the dataset integrates the existing sequences of presently identified SarbeCoVs, along with related RBD and RBM sequences. The dataset offers a platform for users to generate an individual reference library for the identification and characterization of novel SarbeCoVs or associated variants. For instance, considering that the spike protein can bind host receptors and is instrumental in the entry of SarbeCoVs into host cells, researchers might exploit the RBD/RBM region to examine potential interspecies spillover events. Utilizing all known natural reservoirs and intermediate hosts associated with complete SarbeCoV sequences as an example, the homology-based heatmap created by whole genome sequences can provide a straightforward clue as to which virus identified from a specific reservoir/intermediate and location is closely related to SARS-CoV and SARS-CoV-2 (Fig. 2a). Furthermore, phylogenetic analysis based on the S protein sequences provided an overview of the viral population within four clades of SARSr-CoVs (clade 1), SC2r-CoVs (clade 2) and two other SarbeCoVs (clade 3 and 4). For the SC2r-CoVs in clade 2, multiple sequence alignment of representative RBMs (Fig. 2b) revealed that the majority, despite sharing higher sequence identity at the genome level, may not bind to human ACE2 (hACE2) due to intrinsic deletions in the key region^123^. However, three newly identified SC2r-CoVs (BANAL-20-52, BANAL-20-103, and BANAL-20-236) from *Rhinolophus malayanus* and *Rhinolophus pusillus* in Laos were found to have an intact RBM similar to that of SARS-CoV-2. In particular, several critical ACE2-interacting residues were almost identical to those found in the RBM of SARS-CoV-2, indicating that they can bind more efficiently to the hACE2, consistent with the findings of previous studies^11,102^. This can also be applied in homology modelling to evaluate RBM binding affinity with ACE2 from different animals^12^. We recommend that users approach such biological interpretations with caution, as *in silico* results always require further experimental verification.

Finally, this dataset represents a time-bounded survey of research efforts on animal-associated SarbeCoVs. As more related viruses are identified and published, the dataset will continue to be updated regularly to provide the latest and most accurate information. The curation protocol outlined in this study can also be utilized in future mapping efforts for other zoonotic viruses. Given that coronaviruses have high frequencies of recombination throughout the genome^2^, we will gradually extend our study subject to the entire range of animal-associated coronaviruses. Furthermore, we also intend to develop an online platform and integrate a set of online visualization tools for easy browsing, text querying, BLAST searching, phylogenetic reconstruction, and various customized comparative analyses of viral diversity between/within different host species.

## Data Availability

There is no custom code produced during the collection and validation of this dataset.
